# My Voice Capturing My Attention to Myself: The Effects of Objective Self-Awareness on Japanese People

**DOI:** 10.3389/fpsyg.2020.01596

**Published:** 2020-07-16

**Authors:** Asuka Narita, Keiko Ishii

**Affiliations:** ^1^Department of Psychology, Graduate School of Humanities, Kobe University, Kobe, Japan; ^2^Department of Cognitive and Psychological Sciences, Graduate School of Informatics, Nagoya University, Nagoya, Japan

**Keywords:** objective self-awareness, one’s own voice, self-esteem, cheating, Japanese

## Abstract

Previous research has demonstrated that the presence of a mirror does not influence Japanese people’s self-evaluation and cheating behaviors, which reflects their tendency to habitually think of themselves based on their imagined perspectives of others. The present work extends the evidence by manipulating the presence of the participants’ own voices as well as that of a mirror (Study 1); it explores the effects of another participant’s voice (Study 2). Consistent with previous findings, the presence of a mirror does not influence Japanese participants’ self-esteem, moral values, and cheating behaviors. In contrast, an impact of their own voice was partially found. The exposure to their own voice decreased the participants’ moral value of fairness and discouraged the participants from cheating by drawing additional coins. Furthermore, no effect of other voices was found. Overall, we observed a limited effect of self-focusing stimuli in Japanese participants, although it should be noted that their own voices were relatively effective for capturing self-focused attention.

## Introduction

People can experience themselves as a subject and direct their conscious attention externally to the surrounding environment, and they can experience themselves as the object of other people’s attention and direct their conscious attention to themselves. According to objective self-awareness (OSA) theory (Duval and Wicklund, 1972), people have bidirectional conscious attention; this refers to when attention is directed away from the self (called subjective self-awareness) and when attention is directed toward the self [called (OSA)]. Both are mutually exclusive because people cannot direct their attention away from and toward the self at the same time. Thus, people usually go back and forth quickly between subjective self-awareness and OSA. Previous research has demonstrated that when people are presented with a stimulus, such as a mirror and a video camera, that makes them focus on themselves, they are likely to be placed in a state of OSA, to realize their internal standards of correctness, and to find discrepancies between their ideal and actual selves (for a review, see Silvia and Duval, 2001). As a result, when in a state of OSA, people view themselves less positively—because they believe they have fallen short of their own standards (Ickes et al., 1973)—and engage in moral and normative behaviors (e.g., the inhibition of cheating and prosocial behaviors; Diener and Wallbom, 1976; Wegner and Schaefer, 1978).

Currently, little is known about whether culture influences whether people will decrease their positive self-evaluation and engage in moral and normative behaviors when they are presented with a stimulus that turns their attention to themselves. This is because the number of non-Western studies on self-awareness is limited. One exception is a study by Heine et al. (2008), which tested the effect of OSA on Japanese and North American participants who were manipulated by a mirror. To our knowledge, no study has demonstrated evidence on the effect of OSA outside of Western cultures since Heine et al. (2008). Thus, we attempted to fill this gap by examining the effect of OSA on Japanese people and determining how culturally specific views of self influence Japanese people’s reaction to self-focusing stimuli.

Numerous empirical studies have reported cultural differences in how people view the self. The differences have been revealed based on a comparison between independent and interdependent selves. The independent self is common in Western cultural contexts and is characterized as an independent and autonomous entity who is separate from other people, whereas the interdependent self is common in East Asian cultural contexts and is characterized as an entity who is interrelated to and connected with others and inseparable from social contexts (Markus and Kitayama, 1991; Oyserman et al., 2002). The differences in these views of the self are reflected in the extent to which people think they are considered by others and imagine they appear to others. For example, compared to Westerners, Japanese people are more likely to emphasize physical attributes and appearance when describing themselves (Kanagawa et al., 2001). Moreover, compared to Westerners, East Asians are more likely to adopt a third-person perspective when recalling situations in which they were included (Cohen and Gunz, 2002), and they are more attuned to others’ perspectives in a communication game (Wu and Keysar, 2007). These findings suggest that reflecting the interdependent view of the self, East Asians are habitually attentive to the viewpoints of others.

Given East Asians’ chronic need to consider others’ viewpoints, Heine et al. (2008) predicted that individuals’ reactions to self-focusing stimuli differ across cultures. They found that North Americans are less likely to view themselves positively and cheat on tasks when they are exposed to a mirror and can see themselves compared to their counterparts without a mirror. This finding replicated those of previous research (Ickes et al., 1973; Diener and Wallbom, 1976). In contrast, a mirror was found to have no effect on Japanese participants, who acted as if they were always exposed to a mirror. This further highlights East Asians’ chronic need to consider others’ viewpoints.

The present research aimed to further investigate the effect of OSA on Japanese people to better understand self-awareness in East Asian cultures. To do so, we focused on two issues not addressed by Heine et al. (2008). First, although Heine et al. (2008) used a mirror, they did not test the effect of other stimuli that may enhance OSA. Therefore, in addition to a mirror, in the present research, we used participants’ own voices, which were recorded in advance, as a stimulus raising OSA. Using a recording of participants’ own voices is a popular way to activate OSA in experiments (e.g., Wicklund and Duval, 1971; Ickes et al., 1973; Vallacher and Solodky, 1979). More important, as Heine et al. (2008) pointed out, previous attempts to examine the effects of OSA on ratings of self in Japan (Mizuta, 1987, Study 1) failed to find an increase in the number of discrepancies between participants’ ideal self and actual self as a result of OSA being activated by a video camera. In contrast, Mizuta (1987, Study 2) used participants’ own tape-recorded voices to manipulate OSA and demonstrated an increase of discrepancies between participants’ ideal and actual selves. It should be noted that as the Japanese participants in Mizuta (1987, Study 2) read a text from a reflective diary and heard their voices being recorded while reading the text, both their own voices and the reflective content influenced the increase of discrepancies. Despite the confounding effect of the reflective content, however, Mizuta’s (1987, Study 2) findings imply that participants’ own voices may be more effective than a mirror as a self-focusing stimulus for Japanese people. Thus, using recordings of participants’ own voices can be a strict test of the effect of OSA on Japanese people.

Second, although Heine et al. (2008) mentioned that “the self-focused attention that a mirror provides may be different from the kind of attention elicited by the critical glare of schematic faces” (p. 885), they did not contrast the effect of self-focused attention with the effect of attention on the self that can be induced by cues provided by others (e.g., another’s voice). For exploratory purposes, we prepared a new condition in which the participants were presented with another participant’s voice; we then compared the new condition with the own-voice condition. Past studies on OSA treated other participants’ voices as a control stimulus that did not increase self-awareness, and they compared this effect with the effect of one’s own voice (Ickes et al., 1973; Diener and Wallbom, 1976). Diener and Wallbom (1976) demonstrated that people cheated more when they were seated to the side of a mirror and were exposed to another’s voice than when they were seated in front of the mirror and were exposed to their own tape-recorded voice. Moreover, a recent meta-analysis study suggested mixed evidence on the effect of others’ watching eyes on one’s prosocial behaviors (Northover et al., 2017). In addition, the image of others’ watching eyes does not influence individuals’ cheating behaviors across several tasks (Cai et al., 2015). Despite such evidence, East Asians may be influenced by these cues, highlighting their interdependent view of the self. Previous studies demonstrated that when East Asian participants were exposed to cues provided by others (e.g., schematic faces of others), the cues led them to think about other people’s expectations and made them imagine that their choices and judgments were insufficient compared to others’ expectations. They were thus likely to feel concern about themselves and justify their choices and judgments to reduce the concern (Kitayama et al., 2004; Kitayama and Park, 2014). Taken together, previous findings do not paint a clear picture of the effect of cues provided by others on OSA in East Asian cultures. We thus explored the effect of other participants’ voices without any specific expectations.

We conducted two studies by extending Heine et al. (2008) study, which showed no impact of the presence of a mirror on Japanese people. In Study 1, we manipulated the presence of participants’ own voices as well as that of a mirror, and we compared these self-focusing stimulus conditions with a control condition in which neither mirror nor participants’ voices were presented. In Study 2, instead of using a mirror, we explored the effects of other participants’ voices and participants’ own voices. Moreover, because we manipulated the presence of participants’ own voices identically between the two studies, we were able to draw a comparison between the results of the own-voice condition and those of the control condition by combining the two studies. In contrast to Heine et al. (2008), this allowed us to properly evaluate the impact of one’s own voice on Japanese people.

Following Heine et al. (2008), we included a measurement of positive self-views (self-esteem) and of cheating behaviors, although we used a different cognitive task—one developed by Gino et al. (2009)—that enabled participants to earn more money by making false reports of the number of correct answers. Moreover, for exploratory purposes, we added a measurement of moral values (Graham et al., 2009), which consisted of five core foundations (i.e., harm, fairness, loyalty, authority, and sanctity). Given that many people believe cheating to be morally wrong and that individuals exposed to self-focusing stimuli reduce their cheating behaviors (Diener and Wallbom, 1976), participants exposed to self-focusing stimuli might show greater endorsement of these moral values. In contrast, previous research suggested the possibility that people change their internal standards to be consistent with the self when they are self-aware (Silvia and Duval, 2001). In particular, when participants are in a state of OSA and focus on an experimental performance standard that they failed to pass, they tend to evaluate the standard negatively and change the standard to match their bad performance (Dana et al., 1997). Moreover, based on the positive relationship between self-esteem and morality (Campbell et al., 2002), a decrease in positive views of the self caused by self-awareness might be related to a decrease in morality. Taken together, the previous findings do not provide a clear picture of the effect of self-focusing stimuli on moral values, so we explored it without any specific expectation.

In summary, consistent with the findings of Heine et al. (2008), we anticipated that the impact of self-focusing stimuli would be limited in Japanese people, although the impact of their own voices would be relatively observable. Thus, Japanese participants exposed to their own voices might evaluate themselves more negatively and cheat less compared to participants in the control condition. Further, we included the other-person’s-voice condition and the measurement of moral values and explored participants’ reactions to the other’s voice and the effect of self-awareness on moral values.

## Study 1

### Method

Ninety-eight Japanese undergraduates (56 females and 42 males, *M*_age_ = 19.21, *SD* = 1.11) participated in this study. They were recruited for a maximum bonus payment of 1,000 yen (about 10 USD) in addition to a payment of 500 yen (about 5 USD). We tested the participants individually and randomly assigned them to one of three conditions: own voice (18 females and 14 males), mirror (18 females and 14 males), and control (20 females and 14 males). There was no gender effect in the following analyses. We determined the sample size based on Heine et al. (2008), who collected data of 30 or 31 participants per condition.

We escorted participants to an individual cubicle and seated them in front of a desk where a laptop computer and a microphone were set up. They first engaged in a task related to communication in which they were asked to read out paragraphs from a fictional work (“The Second Bakery Attack” by Haruki Murakami) at their usual speaking rate. Different from the contents of the reflective diary used in Mizuta (1987, Study 2), the contents of the paragraphs consisted of a conversation between a husband and wife about the husband’s old friend. We told the participants in advance that their reading would be recorded by the experimenter. They continued reading until the experimenter interrupted them after 5 min. Then, we asked them to perform a filler task on communication for about 20 min for 500 yen. In the filler task, the participants listened to a daily conversation between two speakers and read aloud a text written by one of the speakers.

Next, we asked the participants to move to another individual cubicle, where they were seated in front of a desk with a laptop computer and a 60-by-90 cm mirror. The size of the mirror was identical to that used in Heine et al. (2008). We hung a portable wireless speaker on the door of the cubicle. In the own-voice and control conditions, we flipped the mirror so that the participants could not see themselves. Following the procedure used in Heine et al. (2008), in all the conditions, we told the participants that the mirror had been left there for an unrelated study. We also told them that because the following task intended to examine the influence of marginal sounds on individuals’ performance, some sounds might play while they worked on their task. We asked participants to complete questionnaires and then engage in a cognitive task. In the own-voice condition, we presented participants’ voices recorded in the first communication task while they were filling in the questionnaires and performing the task. In the mirror condition, participants filled in the questionnaires and performed the task in front of the mirror, which enabled them to see themselves. In the control condition, neither the participants’ voices nor their mirror images were presented while they were filling in the questionnaires and performing the task.

One of the two scales included in the questionnaires was the Rosenberg Self-Esteem Scale (Rosenberg, 1965), which consists of 10 items (e.g., On the whole, I am satisfied with myself). We asked participants to rate the extent to which they agreed with each statement using a six-point scale (1 = not at all, 6 = completely). Two Japanese–English bilinguals translated and back-translated the items between Japanese and English to ensure cross-cultural equivalence. The Cronbach’s alpha was 0.86. The other scale was the Moral Foundations Questionnaire (MFQ; Graham et al., 2009), which consisted of 15 moral relevance items and 15 moral judgment items related to five foundations (i.e., three moral relevance items and three moral judgment items for each foundation). For the moral relevance items, we asked the participants to rate using a six-point scale (0 = not at all, 5 = extremely) the extent to which each statement was relevant to their decision about whether something was right or wrong [e.g., “Whether or not someone suffered emotionally” (harm), “Whether or not some people were treated differently than others” (fairness), “Whether or not someone’s action showed love for his or her country” (loyalty), “Whether or not someone showed a lack of respect for authority” (authority), and “Whether or not someone violated standards of purity and decency” (sanctity)]. For the moral judgment items, we asked participants to rate the extent to which they agreed with each statement using a six-point scale (0 = not at all, 5 = strongly) [e.g., “Compassion for those who are suffering is the most crucial virtue” (harm), “When the government makes laws, the number one principle should be ensuring that everyone is treated fairly” (fairness), “I am proud of my country’s history” (loyalty), “Respect for authority is something all children need to learn” (authority), and “People should not do things that are disgusting, even if no one is harmed” (sanctity)]. We used the Japanese translated version (Kanai, 2013) of the MFQ in this study. The Cronbach’s alphas were 0.52 for harm, 0.69 for fairness, 0.48 for loyalty, 0.60 for authority, and 0.48 for sanctity.

Finally, in the cognitive task, participants first received a brown envelope containing 1,000 yen (20 50 yen coins). Their task was to choose two numbers in a matrix presented on the screen of the laptop computer so that the sum of the two numbers was 10. They also received a sheet of paper and a pen so that they could calculate the answers and record the number of correct trials if needed. The task consisted of 20 trials. For each trial, a matrix was presented containing 12 numbers, each indicating two decimal places (e.g., 8.37). After choosing two numbers and clicking a button marked “answer,” the participants could move on to the next trial. We told participants that they had 2 min to complete the task and that they would earn 50 yen if they found two numbers correctly for each matrix and leave unearned money in the envelope on the desk. On the computer screen, they could monitor the remaining time. As soon as the 2 min were up, the participants had to draw coins corresponding to the number of total correct trials from the envelope by themselves. For each trial, although the participants’ chosen answers and the time it took them to find the answers were recorded on the computer to keep track of participants’ behaviors, the participants did not receive any feedback on whether their answers were correct or incorrect. They were allowed to advance to the next trial by clicking the answer button regardless of whether their answers were correct or incorrect. They were also allowed to advance to the next trial if they clicked the answer button without answering a given trial. However, they could not go back to previous trials. Thus, in this situation designed to maximize their motivation for cheating, participants could finish the task without giving any answers and take all the coins. Although some participants might misunderstand the number of correct trials and cheat unintentionally, we expected few participants to do so, because they would be allowed to use a piece of paper and pen to record the number. Even if some participants cheated unintentionally, the probability would not differ across the conditions.

We also told the participants that the experimenter would keep the door of the cubicle closed, go out of the experiment room during the task, and return after they had finished the task. They were also told that the experimenter would not ask them something that could identify them, including their signature for a receipt of payment. In reality, 7 min into the task, an alternative experimenter showed up and opened the door of the cubicle. The alternative experimenter told the participants that the experiment had just ended and that the participants could leave with the money they had earned. Thus, in this situation, the participants were not seen by anyone (or at least by the first experimenter), and it was unclear how many coins they drew from the envelope corresponding to the number of correct trials or how long they engaged in the task. For each trial, the alternative experimenter confirmed that the participant left the experiment room, upon which the first experimenter returned to the room and counted how many coins were left in the envelope.

### Results

#### Self-Esteem

We performed an ANOVA on the mean rating of items measuring self-esteem with one between-subjects variable (condition: own voice, mirror, control). The main effect was significant, *F*(2,95) = 3.63, *p* = 0.03, η*_*p*_*^2^ = 0.07. The participants reported significantly higher self-esteem in the control condition (*M* = 3.76, *SD* = 0.90) than in the own-voice condition (*M* = 3.28, *SD* = 0.63), Tukey’s HSD *p* = 0.03. The rating of self-esteem in the mirror condition (*M* = 3.62, *SD* = 0.69) did not significantly differ from that in the own-voice condition (Tukey’s HSD *p* = 0.17) or from that in the control condition (Tukey’s HSD *p* = 0.70) (see Table 1).

**TABLE 1:** Effects of stimuli (own voice and mirror) on self-esteem, moral values, and cheating behaviors in Study 1.

	Own voice		Mirror		Control	
Variable	*M*	*SD*	*M*	*SD*	*M*	*SD*
Self-esteem	3.28	0.63	3.62	0.69	3.76	0.90
Harm	3.43	0.56	3.44	0.60	3.66	0.62
Fairness	3.09	0.54	3.17	0.73	3.48	0.50
Loyalty	2.44	0.49	2.42	0.60	2.57	0.66
Authority	2.80	0.47	2.59	0.65	2.80	0.61
Sanctity	2.34	0.53	2.22	0.61	2.30	0.55
Time spent after the time limit (s)	63.13	98.97	64.50	118.31	67.85	102.98
Additional coins drawn (log)	0.18	0.37	0.27	0.44	0.33	0.41

#### Moral Foundations

For each of the five moral foundations, we performed an ANOVA on the mean rating of relevance and judgment items with one between-subjects variable (condition). Relevant means are shown in Table 1. We did not find a significant main effect except for fairness [harm: *F*(2,95) = 1.59, *p* = 0.21, η*_*p*_*^2^ = 0.03; fairness: *F*(2,95) = 4.00, *p* = 0.02, η*_*p*_*^2^ = 0.08; loyalty: *F*(2,95) = 0.61, *p* = 0.55, η*_*p*_*^2^ = 0.01; authority: *F*(2,95) = 1.36, *p* = 0.26; η*_*p*_*^2^ = 0.03; and sanctity: *F*(2,95) = 0.34, *p* = 0.71, η*_*p*_*^2^ = 0.01]. Participants reported significantly higher levels of fairness in the control condition (*M* = 3.48, *SD* = 0.50) than in the own-voice condition (*M* = 3.09, *SD* = 0.54), Tukey’s HSD *p* = 0.02. The fairness rating in the mirror condition (*M* = 3.17, *SD* = 0.73) did not significantly differ from that in the own-voice condition (Tukey’s HSD *p* = 0.84) or from that in the control condition (Tukey’s HSD *p* = 0.09).

#### Cognitive Task

First, we analyzed the number of correct trials solved within the time limit using a one-way ANOVA. The main effect of the condition was not significant, *F*(2,95) = 0.46, *p* = 0.64, η*_*p*_*^2^ = 0.01 (own voice: *M* = 4.47, *SD* = 1.97; mirror: *M* = 4.06, *SD* = 1.83; control: *M* = 4.12, *SD* = 1.75). This suggests that participants were motivated to work on the cognitive task equally across the conditions.

We focused on two measurements of cheating behaviors. One was the amount of time the participants continued to engage in the task after the time limit. Because the time it took to find answers for each trial was recorded, we measured the amount of time each participant spent until giving the last answer after the allowed time limit and performed an ANOVA on that amount of time with one between-subjects variable (condition). There was no significant effect of the condition, *F*(2,95) = 0.02, *p* = 0.98, η*_*p*_*^2^ = 0.00. The other measurement was how many more coins the participants drew than they should have drawn corresponding to the number of correct trials. Because the number of the additional coins was positively skewed, we log-transformed them for each participant [*N*’ = log (*N* + 1), *N* = the number of the additional coins]. The ANOVA showed no significant effect of the condition, *F*(2,95) = 1.03, *p* = 0.36, η*_*p*_*^2^ = 0.02. The proportion of people who cheated by drawing additional coins was 41% (13 out of 32) in the own-voice condition, 44% (14 out of 32) in the mirror condition, and 56% (19 out of 34) in the control condition, χ^2^(2, *N* = 98) = 1.74, *p* = 0.42. Relevant means are shown in Table 1.

### Discussion

Overall, the mirror had no influence on participants’ ratings of self-esteem, moral values, and cheating behaviors. This is consistent with Heine et al.’s findings (2008). In contrast, participants’ own voices had an influence on their ratings of self-esteem and on one of the moral values (fairness). In line with the typical reactions to a self-focusing stimulus, for Japanese participants exposed to their own voices, their self-esteem decreased compared to participants who were not exposed to their own voices. Moreover, corresponding to the pattern of self-esteem, fairness decreased in participants exposed to their own voices. In Study 2, we continued to examine the effect of own voices as well as that of other participants’ voices, which were used instead of a mirror for exploratory purposes.

## Study 2

### Method

Sixty-two Japanese undergraduates (38 females and 24 males, *M*_age_ = 19.82, *SD* = 1.33) participated in the study. They were recruited for a maximum bonus payment of 1,000 yen (about 10 USD) in addition to a payment of 500 yen (about 5 USD). We tested the participants individually and randomly assigned them to one of three conditions: own voice (13 females and eight males), other voice (13 females and eight males), and control (12 females and eight males). The sample size was initially determined in the same way as in Study 1 (i.e., 30 or more per condition). However, we could not collect enough data by the end of the semester, when the first author had to submit an undergraduate thesis based on this study to graduate university and obtain a bachelor’s degree. There was no gender effect in the following analyses.

This procedure was similar to that used in Study 1 except in two ways: First, instead of the effect of a mirror, we explored the effect of others’ voices on individuals’ self-esteem, morality, and cheating behaviors. In the other-voice condition, we presented the participants with the voice of another same-sex participant while they were filling in the self-esteem and moral foundation questionnaires and performing the cognitive task. Second, we removed the mirror used in Study 1 in all the Study 2 conditions. Thus, the experimenter did not mention anything related to the mirror in the instruction.

### Results

#### Self-Esteem

The Cronbach’s alpha was 0.88. The ANOVA of the average rating of items measuring self-esteem with one between-subjects variable (condition: own voice, other voice, control) showed no significant effect of the condition, *F*(2,59) = 0.11, *p* = 0.90, η*_*p*_*^2^ = 0.00. Relevant means are presented in Table 2.

**TABLE 2:** Effects of stimuli (own voice and other voice) on self-esteem, moral values, and cheating behaviors in Study 2.

	Own voice		Other voice		Control	
Variable	*M*	*SD*	*M*	*SD*	*M*	*SD*
Self-esteem	3.64	0.81	3.67	0.81	3.56	1.02
Harm	3.56	0.56	3.63	0.73	3.74	0.48
Fairness	3.25	0.58	3.52	0.46	3.36	0.50
Loyalty	2.59	0.49	2.52	0.76	2.67	0.79
Authority	2.96	0.55	2.64	0.59	2.76	0.88
Sanctity	2.48	0.50	2.44	0.55	2.45	0.69
Time spent after the time limit (s)	61.48	98.66	72.67	107.45	97.70	129.68
Additional coins drawn (log)	0.11	0.30	0.34	0.44	0.37	0.41

#### Moral Foundations

The Cronbach’s alphas were 0.61 for harm, 0.44 for fairness, 0.53 for loyalty, 0.66 for authority, and 0.46 for sanctity. For each of the five moral foundations, we performed an ANOVA on the average rating of items with one between-subject variable (condition) as in Study 1. We did not find a significant main effect [harm: *F*(2,59) = 0.46, *p* = 0.63, η*_*p*_*^2^ = 0.02; fairness: *F*(2,59) = 1.55, *p* = 0.22, η*_*p*_*^2^ = 0.05; loyalty: *F*(2,59) = 0.23, *p* = 0.80, η*_*p*_*^2^ = 0.01; authority: *F*(2,59) = 1.16, *p* = 0.32, η*_*p*_*^2^ = 0.04; and sanctity: *F*(2,59) = 0.02, *p* = 0.98, η*_*p*_^2^* = 0.00]. The relevant means are also presented in Table 2.

#### Cognitive Task

As in Study 1, we first analyzed the number of correct trials solved within the time limit using a one-way ANOVA. The main effect of the condition was significant, *F*(2,59) = 4.51, *p* = 0.02, η*_*p*_*^2^ = 0.13. The participants in the own-voice condition solved more trials correctly (*M* = 4.76, *SD* = 2.07) than did those in the control condition (*M* = 3.00, *SD* = 1.89), Tukey’s HSD *p* = 0.02. This trend is consistent with Wicklund and Duval (1971, Study 3), demonstrating that OSA facilitates task performance. In contrast, the number of correct trials in the other-voice condition (*M* = 4.43, *SD* = 1.99) did not differ from that in the own-voice condition (Tukey’s HSD *p* = 0.85) or the control condition (Tukey’s HSD *p* = 0.06). We thus included the number of correct trials solved within the time limit as a covariate in the following analyses on cheating behaviors.

We computed the amount of time that each participant engaged in the task after 2 min had passed and the number of additional coins she or he drew and analyzed the measured cheating behaviors in the same way as in Study 1. Because the number of additional coins was positively skewed, we log-transformed them for each participant as we did in Study 1. ANCOVAs showed that the main effect of the condition was not significant for either the measurement of the amount of time, *F*(2,58) = 0.55, *p* = 0.58, η*_*p*_*^2^ = 0.02, or the measurement of additional coins, *F*(2,58) = 2.95, *p* = 0.06, η*_*p*_*^2^ = 0.09^1^. The proportion of people who cheated by drawing additional coins was 38% (eight out of 21) in the own-voice condition, 62% (13 out of 21) in the other-voice condition, and 65% (13 out of 20) in the control condition, χ^2^(2, *N* = 62) = 3.63, *p* = 0.16. The relevant means are shown in Table 2. Interestingly, the number of correct trials solved within the time limit significantly influenced the measurement of the additional coins, *F*(1,58) = 3.92, *p* = 0.05, η*_*p*_*^2^ = 0.06. Overall, participants who solved more trials correctly drew fewer additional coins.

### Discussion

Own voice had no significant influence on participants’ ratings of self-esteem, moral values, and cheating behaviors. Additionally, we did not find an effect of the other-voice condition. This suggests that cues from other people that activate one’s perception of their expectations do not affect cheating behaviors. This is consistent with the previous research by Cai et al. (2015).

## Comparison Between the Own-Voice Condition and the Control Condition

One limitation of this research was that both studies could be considered underpowered because of the relatively small sample sizes. To address this limitation, we drew a comparison between the own-voice condition (*N* = 53) and the control condition (*N* = 54) by combining the two studies. We conducted both studies using the same procedure. This sample size was in accordance with the one computed based on a value for desired power of 0.80 and on a medium effect size (*d* = 0.55).

### Results and Discussion

#### Self-Esteem

The effect of the condition on self-esteem was not significant, *t*(105) = 1.64, *p* = 0.10, *d* = 0.32 (own-voice condition: *M* = 3.42, *SD* = 0.72; control condition: *M* = 3.69, *SD* = 0.94).

#### Moral Foundations

Whereas participants in the own-voice condition (*M* = 3.15, *SD* = 0.56) showed significantly lower levels of fairness than those in the control condition (*M* = 3.44, *SD* = 0.50), *t*(105) = 2.79, *p* = 0.006, *d* = 0.54, we did not find a significant effect of the condition on the other moral foundations [harm: *t*(105) = 1.91, *p* = 0.06, *d* = 0.37; loyalty: *t*(105) = 0.89, *p* = 0.38, *d* = 0.17; authority: *t*(105) = 0.68, *p* = 0.50, *d* = 0.13; and sanctity: *t*(105) = -0.33, *p* = 0.74, *d* = 0.06].

#### Cognitive Task

The participants in the own-voice condition solved more trials correctly within the time limit (*M* = 4.58, *SD* = 1.99) than those in the control condition (*M* = 3.70, *SD* = 1.87), *t*(105) = 2.36, *p* = 0.02, *d* = 0.46. Controlling the effect of the number of correct trials solved within the time limit, we performed ANCOVAs on the two measurements of cheating behaviors. Although there was no significant difference in the amount of time that participants worked on the task after the 2 min had passed between the own-voice condition (*M* = 62.47, *SD* = 97.90) and the control condition (*M* = 78.91, *SD* = 113.33), *F*(1,104) = 0.68, *p* = 0.41, η*_*p*_*^2^ = 0.01, the main effect of the covariate was significant, *F*(1,104) = 6.66, *p* = 0.01, η*_*p*_*^2^ = 0.06. Participants who solved more trials correctly worked less on the cognitive task after the time limit. At the same time, the influence of participants’ own voices was found to be significant in the log-transformed measurement of additional coins, *F*(1,104) = 6.80, *p* = 0.01, η*_*p*_*^2^ = 0.06, whereas the main effect of the covariate was not found to be significant, *F*(1,104) = 2.84, *p* = 0.10, η*_*p*_*^2^ = 0.03. Participants in the own-voice condition (*M* = 0.15, *SD* = 0.34) drew fewer additional coins than those in the control condition (*M* = 0.34, *SD* = 0.40)^2^. Furthermore, we calculated the proportion of people who drew additional coins. In the control condition, 59% (32 out of 54) of the participants drew additional coins. The proportion dropped significantly to 40% (21 out of 53) in the own-voice condition, χ^2^(1, *N* = 107) = 4.13, *p* = 0.04.

In summary, the effect of own voice was partly observed when we drew a comparison between the own-voice condition and the control condition by combining the two studies. Fairness decreased in the Japanese participants who were exposed to their own voices. Meanwhile, we did not find an effect of the own-voice condition on self-esteem and the other domains of moral values. Further, exposure to own voice led Japanese participants to reduce their motivation to cheat and draw additional coins. The participants who were exposed to their own voice solved more trials correctly within the time limit than those in the control condition. Finally, the participants with a larger number of correct trials within the time limit worked less on the task after the time limit.

## General Discussion

We found that in Study 1, the presence of the mirror did not influence Japanese participants’ self-esteem, moral values, and cheating behaviors, in line with Heine et al.’s results (2008). In contrast, an impact of participants’ own voices was partially found. In Study 1, those exposed to their own voices evaluated self-esteem and fairness as lower than those in the control condition. In Study 2, neither own voice nor other voice influenced the measurements of self-esteem, moral values, and cheating behaviors. In the analysis of the combined data of the two studies to find potential differences between the own-voice condition and the control condition, the presence of their own voice increased participants’ efforts to perform well and discouraged them from cheating by drawing more coins than they should have. It also decreased participants’ value of fairness. To our knowledge, this is the first piece of evidence of its kind in the literature, because Heine et al. (2008) did not address the effect of participants’ own voices, and Mizuta (1987) did not focus on either moral values or cheating behaviors.

As Heine et al. (2008) argued, the results can be interpreted based on participants’ interdependent view of the self. This self-view leads Japanese people to habitually view themselves based on their imagined perspectives of others. As a result, Japanese people are chronically in a state of OSA, as if they always have a mirror in their heads. However, the results also suggest that compared to a mirror, their own voice is stronger as a self-focusing stimulus. Thus, Japanese people presented with their own voice would become further self-aware and consider further others’ viewpoints. Accordingly, their performance would be further enhanced, and their cheating behaviors such as taking money would be further suppressed.

Why was hearing one’s own voice relatively effective for enhancing self-awareness even among Japanese participants? Whereas participants could consciously avoid seeing themselves in the mirror (although they would pay spontaneous attention to themselves as if they were being seen by others), their own voice could capture self-focused attention because they would find it impossible to cover their ears with their hands while engaging in the tasks. Further, Japanese people are more likely than Westerners to be attuned to vocal processing (Ishii et al., 2003; Tanaka et al., 2010)—reflecting their high-context communication style (Hall, 1976)—causing the impact of their own voice to be relatively greater.

The analysis of the combined data of the two studies showed that the presence of own voice led to not only an inhibition regarding taking more money but also a decrease in fairness. Additional analysis showed that there was no significant correlation between the rating of fairness and the number of additional coins drawn in either the own-voice condition (*r* = -0.20, *p* = 0.15) or the control condition (*r* = -0.08, *p* = 0.57). Both are thus considered to be independent measurements. Taken together, one conjecture is that when in a state of OSA, while people might become more aware of their ideal self and internal standards of correctness and work harder to meet the standards (i.e., changing self), they might also evaluate their standards of fairness negatively to match their actual and negatively evaluated self (i.e., changing standards). Changing self and changing standards, which appear independently, reflect an individual’s motivation to reduce the discrepancy in self and standards. However, it should be noted that the level of internal consistency for MFQ was relatively low. A recent study by Murayama and Miura (2019) investigating the validity of the Japanese version of the MFQ with a larger Japanese sample (*N* = 855) also reported relatively low Cronbach’s alpha coefficients (0.69 for harm, 0.63 for fairness, 0.57 for loyalty, 0.57 for authority, and 0.56 for sanctity). Additionally, because there was no significant correlation between the ratings of self-esteem and fairness in either the own-voice condition (*r* = 0.14, *p* = 0.30) or the control condition (*r* = -0.01, *p* = 0.94), previous findings suggesting a positive relationship between self-esteem and morality (Campbell et al., 2002) were not supported. These things may make it difficult to interpret why the presence of own voice decreases the value of fairness, whereas it suppresses cheating behaviors. Although we added the measurement of moral values for an exploratory purpose, further work should be conducted to address the validity of the current findings on the relationships among OSA, the moral value of fairness, and cheating behaviors using a better method for assessing morality.

It should be noted that although the effect of exposure on the participants’ own voices was recognized as mentioned above, it had only a few clear consequences for their self-evaluations and cheating behaviors. Given that manipulating participants’ own voices was considered a strict test of the effect of OSA on Japanese people, the partial effect of their own voices reported in this research suggests the difficulty of replicating OSA effects for Japanese people. Thus, the assumption that Japanese people are chronically in a state of OSA is highly credible. Because this assumption is based on an orientation toward interdependence—which is emphasized in East Asian cultures—it can be applied to other East Asian cultures as well. Future work will be needed in other East Asian cultures using manipulations of both mirrors and own voices to investigate OSA effects. The findings will advance research on the relationship between culture and self-awareness.

Study 2 explored the effect of other voice, which was not addressed by Heine et al. (2008). Whereas previous research demonstrated that cues provided by others (e.g., schematic faces of others) influenced Japanese people’s views of the interdependent self (Kitayama et al., 2004), other voice has been treated as a low self-focusing stimulus in the literature on OSA (Ickes et al., 1973; Diener and Wallbom, 1976). Additionally, previous findings indicated that the image of watching eyes had no effect on cheating behaviors (Cai et al., 2015). The current findings are consistent with the previous findings on OSA. However, because of Study 2’s null result, this research could not distinguish between the consequence of self-focused attention evoked by participants’ own voices and the self-focused attention elicited by cues provided by others (e.g., other voice). Previous research suggests that the attention evoked by a mirror and by participants’ own voices is likely directed toward private and covert aspects of the self, whereas audiences and TV cameras likely capture individuals’ attention to the public and social aspects of the self (Scheier et al., 1979). Further, participants’ responses vary depending on which aspects of the self are activated by the self-focusing stimuli. For instance, although both types of attentions are associated with dissonance reduction, participants exposed to a mirror are likely to reduce dissonance by misperceiving their counter-attitudinal behavior (and not by changing their attitudes), whereas participants exposed to a camera are likely to reduce dissonance by changing their attitudes (and not by perceiving their behavior as counter-attitudinal) (Scheier and Carver, 1980). It is important that future work investigates whether and to what extent the two types of attention are evoked by different stimuli and that the consequences are differentiated in other cultural contexts, particularly in East Asian ones, as suggested by Scheier and Carver.

The present research has some limitations. One limitation is that the sample size of each study was relatively small. Thus, we found some inconsistencies between the studies. For instance, whereas participants exposed to their own voice evaluated themselves less positively than did those in the control condition in Study 1, this tendency disappeared in Study 2. Moreover, because of the relatively small sample size, we were unable to detect the effect of the mirror in Study 1 and the effect of other voice in Study 2, compared to the control condition. With regard to the sample size issue, our findings revealed the effect of own voice on taking money and on fairness, based on an analysis of the combined data of the two studies. Again, the results support the proposition that Japanese people are chronically in a state of OSA and also suggest that hearing their own voice likely makes Japanese people further self-aware.

Another limitation is that we focused only on Japanese people. Even if we know the effect of own voice as well as the effect of a mirror on Westerners, future work that directly looks at the effect of own voice across cultures will be needed. Another limitation relates to how self-awareness was measured. In line with previous research, we measured individuals’ reactions to self-focusing stimuli and evaluated the occurrence of OSA based on this. Thus, the weak effects of self-focusing stimuli for Japanese people may result from the indirect measurement of OSA. By using neuroscience measures such as functional magnetic resonance imaging, future work may be able to observe the OSA effects induced by self-focusing stimuli more directly and examine the underlying brain processes.

Despite these limitations, the present research, which has suggested the difficulty of replicating OSA effects for Japanese people, contributes to our understanding of the significance of cultural contexts for the conception of the self. Moreover, this research sheds light on the differentiation of OSA effects caused by different types of self-focusing stimuli. Given the problem of generalizing findings from WEIRD (Western, educated, industrialized, rich, and democratic) people, as Henrich et al. (2010) pointed out, it is important to investigate, in a more elaborate way, the OSA effects for people in non-Western cultural contexts and to elucidate the nature of culturally sanctioned forms of the self.

## Data Availability Statement

The datasets generated for this study are available on request to the corresponding author.

## Ethics Statement

The studies involving human participants were reviewed and approved by the Experimental Research Ethics Committee at the Graduate School of Humanities, Kobe University. The patients/participants provided their written informed consent to participate in this study.

## Author Contributions

AN and KI designed the research, analyzed the data, and wrote the manuscript. AN performed the research. Both authors contributed to the article and approved the submitted version.

## Conflict of Interest

The authors declare that the research was conducted in the absence of any commercial or financial relationships that could be construed as a potential conflict of interest.

## Footnotes

1 The main effect of the condition was not significant for either the measurement of the amount of time, *F*(2,59) = 0.56, *p* = 0.58, η_*p*_*^2^* = 0.02, or the log-transformed measurement of additional coins, *F*(2,59) = 2.81, *p* = 0.07, η_*p*_*^2^* = 0.09, even when we performed an ANOVA by excluding the covariate (i.e., the number of correct trials solved within the time limit).

2 Even when we performed an ANOVA by excluding the covariate (i.e., the number of correct trials solved within the time limit), the main effect of the condition was not significant for the measurement of the amount of time, *F*(1,105) = 0.64, *p* = 0.42, η*_*p*_*^2^ = 0.01, whereas it was significant for the log-transformed measurement of additional coins, *F*(1,105) = 6.68, *p* = 0.01, η*_*p*_*^2^ = 0.06.

## References

[B1] Cai W. Huang X. Wu S. Kou Y. (2015). Dishonest behavior is not affected by an image of watching eyes. *Evol. Hum. Behav.* 36 110–116. 10.1016/j.evolhumbehav.2014.09.007

[B2] Campbell W. K. Rudich E. A. Sedikides C. (2002). Narcissism, self-esteem, and the positivity of self-views: two portraits of self-love. *Pers. Soc. Psychol. Bull.* 28 358–368. 10.1177/0146167202286007

[B3] Cohen D. Gunz A. (2002). As seen by the other…: perspectives on the self in the memories and emotional perceptions of Easterners and Westerners. *Psychol. Sci.* 13 55–59. 10.1111/1467-9280.00409 11892778

[B4] Dana E. R. Lalwani N. Duval T. S. (1997). Objective self-awareness and focus of attention following awareness of self-standard discrepancies: changing self or changing standards of correctness. *J. Soc. Clin. Psychol.* 16 359–380. 10.1521/jscp.1997.16.4.359

[B5] Diener E. Wallbom M. (1976). Effects of self-awareness on antinormative behavior. *J. Res. Pers.* 10 107–111. 10.1016/0092-6566(76)90088-x

[B6] Duval S. Wicklund R. A. (1972). *A Theory of Objective Self Awareness.* New York, NY: Academic Press.

[B7] Gino F. Ayal S. Ariely D. (2009). Contagion and differentiation in unethical behavior: the effect of one bad apple on the barrel. *Psychol. Sci.* 20 393–398. 10.1111/j.1467-9280.2009.02306.x 19254236

[B8] Graham J. Haidt J. Nosek B. A. (2009). Liberals and conservatives rely on different sets of moral foundations. *J. Pers. Soc. Psychol.* 96 1029–1046. 10.1037/a0015141 19379034

[B9] Hall E. T. (1976). *Beyond Culture.* New York, NY: Doubleday.

[B10] Heine S. J. Takemoto T. Moskalenko S. Lasaleta J. Henrich J. (2008). Mirrors in the head: cultural variation in objective self-awareness. *Pers. Soc. Psychol. Bull.* 34 879–887. 10.1177/0146167208316921 18453391

[B11] Henrich J. Heine S. J. Norenzayan A. (2010). The weirdest people in the world? *Behav. Brain Sci.* 33 61–135. 20550733 10.1017/S0140525X0999152X

[B12] Ickes W. J. Wicklund R. A. Ferris C. B. (1973). Objective self awareness and self esteem. *J. Exp. Soc. Psychol.* 9 202–219.

[B13] Ishii K. Reyes J. A. Kitayama S. (2003). Spontaneous attention to word content versus emotional tone: differences among three cultures. *Psychol. Sci.* 14 39–46. 10.1111/1467-9280.01416 12564752

[B14] Kanagawa C. Cross S. E. Markus H. R. (2001). Who am I?” The cultural psychology of the conceptual self. *Pers. Soc. Psychol. Bull.* 27 90–103. 10.1177/0146167201271008

[B15] Kanai R. (2013). *The Origin of Morality in the Brain.* Tokyo: Iwanami Shoten.

[B16] Kitayama S. Park J. (2014). Error-related brain activity reveals self-centric motivation: culture matters. *J. Exp. Psychol.* 143 62–70. 10.1037/a0031696 23398181

[B17] Kitayama S. Snibbe A. C. Markus H. R. Suzuki T. (2004). Is there any “free” choice? Self and dissonance in two cultures. *Psychol. Sci.* 15 527–533. 10.1111/j.0956-7976.2004.00714.x 15270997

[B18] Markus H. R. Kitayama S. (1991). Culture and the self: implications for cognition, emotion, and motivation. *Psychol. Rev.* 98 224–253. 10.1037/0033-295x.98.2.224

[B19] Mizuta K. (1987). The effect of objective self-awareness on self-evaluation. *Jpn. J. Exp. Soc. Psychol.* 27 59–67. 10.2130/jjesp.27.59 28548501

[B20] Murayama A. Miura A. (2019). Validation of the Japanese version of the Moral Foundation Questionnaire: investigating the relationship with ideologies. *Jpn. J. Psychol.* 90 156–166. 10.4992/jjpsy.90.17234

[B21] Northover S. B. Pedersen W. C. Cohen A. B. Andrews P. W. (2017). Artificial surveillance cues do not increase generosity: two meta-analyses. *Evol. Hum. Behav.* 38 144–153. 10.1016/j.evolhumbehav.2016.07.001

[B22] Oyserman D. Coon H. M. Kemmelmeier M. (2002). Rethinking individualism and collectivism: evaluation of theoretical assumptions and meta-analyses. *Psychol. Bull.* 128 3–72. 10.1037/0033-2909.128.1.3 11843547

[B23] Rosenberg M. (1965). *Society and the Adolescent Self-Image.* Princeton, NJ: Princeton University Press.

[B24] Scheier M. F. Carver C. S. (1980). Private and public self-attention, resistance to change, and dissonance reduction. *J. Pers. Soc. Psychol.* 39 390–405. 10.1037/0022-3514.39.3.390

[B25] Scheier M. F. Carver C. S. Gibbons F. X. (1979). Self-directed attention, awareness of bodily states, and suggestibility. *J. Pers. Soc. Psychol.* 37 1576–1588. 10.1037/0022-3514.37.9.1576 501522

[B26] Silvia P. J. Duval T. S. (2001). Objective self-awareness theory: recent progress and enduring problems. *Pers. Soc. Psychol. Rev.* 5 230–241. 10.1207/s15327957pspr0503_4

[B27] Tanaka A. Koizumi A. Imai H. Hiramatsu S. Hiramoto E. de Gelder B. (2010). I feel your voice: cultural differences in the multisensory perception of emotion. *Psychol. Sci.* 21 1259–1262. 10.1177/0956797610380698 20713633

[B28] Vallacher R. R. Solodky M. (1979). Objective self-awareness, standards of evaluation, and moral behavior. *J. Exp. Soc. Psychol.* 15 254–262. 10.1016/0022-1031(79)90036-2

[B29] Wegner D. M. Schaefer D. (1978). The concentration of responsibility: an objective self-awareness analysis of group size effects in helping situations. *J. Pers. Soc. Psychol.* 36 147–155. 10.1037/0022-3514.36.2.147

[B30] Wicklund R. A. Duval S. (1971). Opinion change and performance facilitation as a result of objective self-awareness. *J. Exp. Soc. Psychol.* 7 319–342. 10.1016/0022-1031(71)90032-1

[B31] Wu S. Keysar B. (2007). The effect of culture on perspective taking. *Psychol. Sci.* 18 600–606. 10.1111/j.1467-9280.2007.01946.x 17614868

